# Mutational analysis of *ARSB* gene in mucopolysaccharidosis type VI: identification of three novel mutations in Iranian patients

**DOI:** 10.22038/IJBMS.2018.27742.6760

**Published:** 2018-09

**Authors:** Nasrin Malekpour, Rahim Vakili, Tayebeh Hamzehloie

**Affiliations:** 1 Department of Human Genetics, School of Medicine, Mashhad University of Medical Sciences, Mashhad, Iran; 2 Division of Endocrinology and Metabolism, Department of Pediatrics, Imam Reza Hospital, Mashhad University of Medical Sciences, Mashhad, Iran

**Keywords:** ARSB gene, Arylsulfatase B, Consanguineous marriage DNA sequencing, Maroteaux-Lamy syndrome, Mucopolysaccharidosis VI (MPS VI)

## Abstract

**Objective(s):**

Mucopolysaccharidosis VI (MPS VI) or Maroteaux-Lamy syndrome is a rare metabolic disorder, resulting from the deficient activity of the lysosomal enzyme arylsulfatase B (ARSB). The enzymatic defect of ARSB leads to progressive lysosomal storage disorder and accumulation of glycosaminoglycan (GAG) dermatan sulfate (DS), which causes harmful effects on various organs and tissues and short stature. To date, more than 160 different mutations have been reported in the *ARSB* gene.

**Materials and Methods:**

Here, we analyzed 4 Iranian and 2 Afghan patients, with dysmorphism indicating MPS VI from North-east Iran. To validate the patients’ type of MPS VI, urine mucopolysaccharide and leukocyte ARSB activity were determined. Meanwhile, genomic DNA was amplified for all 8 exons and flanking intron sequences of the *ARSB* gene to analyze the spectrum of mutations responsible for the disorder in all patients.

**Results:**

Abnormal excretion of DS and low leukocyte ARSB activity were observed in the urine samples of all 6 studied patients. In direct DNA sequencing, we detected four different homozygous mutations in different exons, three of which seem not to have been reported previously: p.H178N, p.H242R, and p.*534W. All three novel substitutions were found in patients with Iranian breed. We further detected the IVS5+2T>C mutation in Afghan siblings and four different homozygous polymorphisms, which have all been observed in other populations.

**Conclusion:**

results indicated that missense mutations were the most common mutations in the *ARSB* gene, most of them being distributed throughout the *ARSB* gene and restricted to individual families, reflecting consanguineous marriages.

## Introduction

Mucopolysaccharidosis VI (MPS VI) or Maroteaux-Lamy syndrome is a progressive autosomal recessive congenital lysosomal storage disorder. At first, Maroteaux *et al.* (1963) described this disorder as a novel dysostosis with increased urinary excretion of chondroitin sulfate (1, 2). Various severe manifestations, including macrocephaly, corneal opacity, facial dysmorphism, degenerative joint disease, chest deformity, umbilical hernias, short stature, and heart defects, affect the quality and longevity of the patient’s life. The patients usually die due to airway obstruction and cardiac defects in the late childhood or adulthood (3-5). The live births incidence of MPS VI is between 1:5000 in the Monte Santo County in Northeast Brazil and 1:1,505,160 births in Sweden (6, 7) and it is estimated that approximately 1,100 persons suffer from MPS VI worldwide, far fewer of which are diagnosed. Brooks *et al.* (1991) characterized the residual N-acetylgalactosamine 4-sulfatase (arylsulfatase B) in fibroblasts from 16 MPS VI patients (8). This enzyme is involved in the catabolism of glycosaminoglycan (GAG) dermatan sulfate (DS) (9). Deficient activity of arylsulfatase B (ARSB) leads to progressive accumulation of DS and affects connective tissues of the skin, heart valves, airways, and the skeleton (6, 10). 

Diagnosis of MPS VI requires clinical phenotype and urinary GAG >100 μg/mg creatinine in cultured fibroblasts or isolated leukocytes. Also, the differential diagnosis should be performed for multiple sulfatase deficiencies, including MPS I, II, IVA, VII, sialidosis, and mucolipidosis (11, 12). Although today enzyme replacement therapy (ERT) with galsulfase (Naglazyme^®^) has provided a better quality of medical care, Naglazyme^®^ is unable to cross the blood-brain barrier and reach the CNS or cornea. Therefore, prevention of affected births through prenatal genetic analysis is still a necessary strategy for high-risk families (13).

The mutations in the *ARSB* gene, on chromosome 5q14.1, is related to the MPS VI disorder. This gene spans 206 kb, comprising 8 exons that generate a 533 amino acid glycoprotein. Until now, around 160 different causative mutations have been detected in the *ARSB* gene, most of which are missense and nonsense mutations while others include small deletions, splice-site mutations, and insertions (14-16). Also, an intragenic deletion in the entire exon 4 of *ARSB* has been detected in a patient with severe MPS VI (17). Most mutations in the *ARSB *gene are rare and family specific. In a study on nine Indian MPS VI patients, researchers reported that each of p.G38-G40del3, p.C91R, p.L98R, and p.R315P mutations were present in one family (18). Moreover, the frequency of some specific variants has been shown in some studies. For instance, c.1143-1G>C has been shown to account for 21.9% of mutant alleles in 16 Spanish and Argentinian MPS VI patients (19). Also, p.L321P mutation and p.V358M polymorphism are detected as the most frequent genetic changes in 13 unrelated Turkish families with MPS VI (7). However, little is known about other mutations and their relevance to the disease clinical outcomes. In the present study, the molecular analysis of six MPS VI patients from 5 unrelated families from the North East of Iran was investigated. Our results led to the identification of three novel substitutions. We also carried out bioinformatic studies to reveal the impact of the observed substitutions on the structure, function, stability, and pathogenicity of the ARSB protein.

## Materials and Methods

A total of 6 patients (P1-P6) including 4 males and 2 females from 5 different families (F1–F5), were clinically diagnosed with MPS VI in Emam Reza Hospital in Mashhad, from 2010-2016. Two of the patients were siblings with Afghan ethnicity.

The information for clinical examinations and radiological and laboratory data were obtained from the patients’ medical records. In this series of patients, the age at onset of symptoms was around 2 to 3 years of age with different manifestations including, vomiting with a high fever, sudden lateral deviation of the eyes and thickening of the bones. In all patients, ARSB activity in leukocytes and excessive excretion of DS in urine was investigated. A definite diagnosis was made on the detection of DS in urine, the deficiency of the relevant enzymatic assay, and the presence of the pathogenic mutation. Informed consent with the appropriate ethics review committee approvals was obtained for each patient and patient’s family. Thereafter, samples were collected for genetic testing.

**Figure 1:**
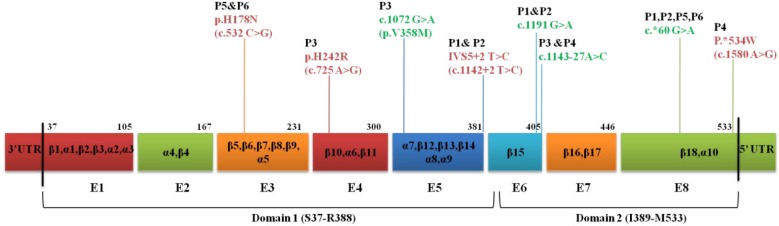
Schematic representation of mutations and polymorphisms in the ARSB gene detected in the patients through this study. The mutations and polymorphisms have been shown with red and green colors, respectively. The exons are represented by colored boxes and numbered by E1 to E8

**Figure 2:**
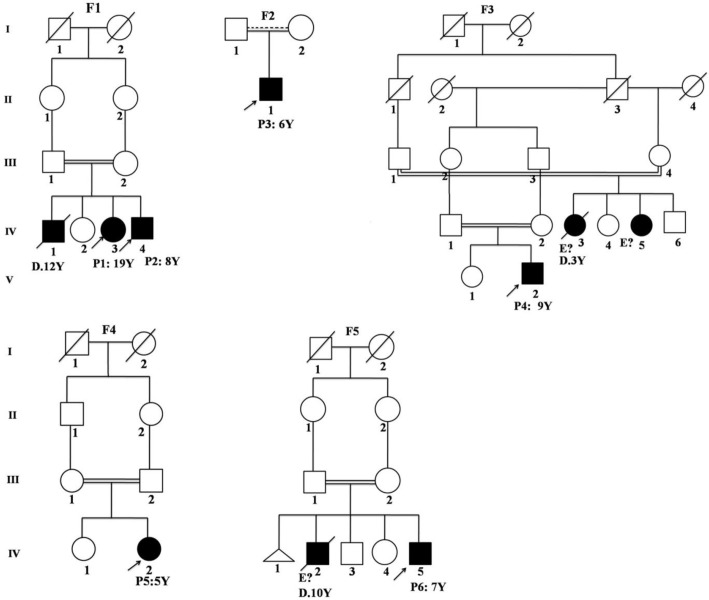
Pedigree of 5 kindreds detected with ARSB deficiency. Each kindred is designated by F1-F5, each generation is designated by a Roman numeral (I – IV), and each individual is designated by an Arabic numeral (from left to right). ARSB deficient patients with a clinical phenotype are represented as closed symbols. In each family, the proband is indicated by an arrow. In the pedigree, individuals whose genetic status could not be evaluated are indicated by “ E?”, thought to be ARSB deficient based on their clinical phenotypes, “P”= patient; “Y”= year; “D”=death, respectively

**Figure 3:**
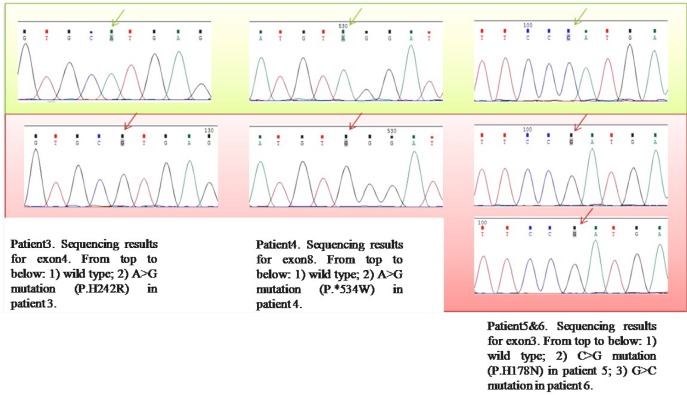
*ARSB* mutation analysis in P3, P4, P5, and P6. Wild-type sequence and novel detected substitutions have been shown with green and red backgrounds, respectively

**Figure 4:**
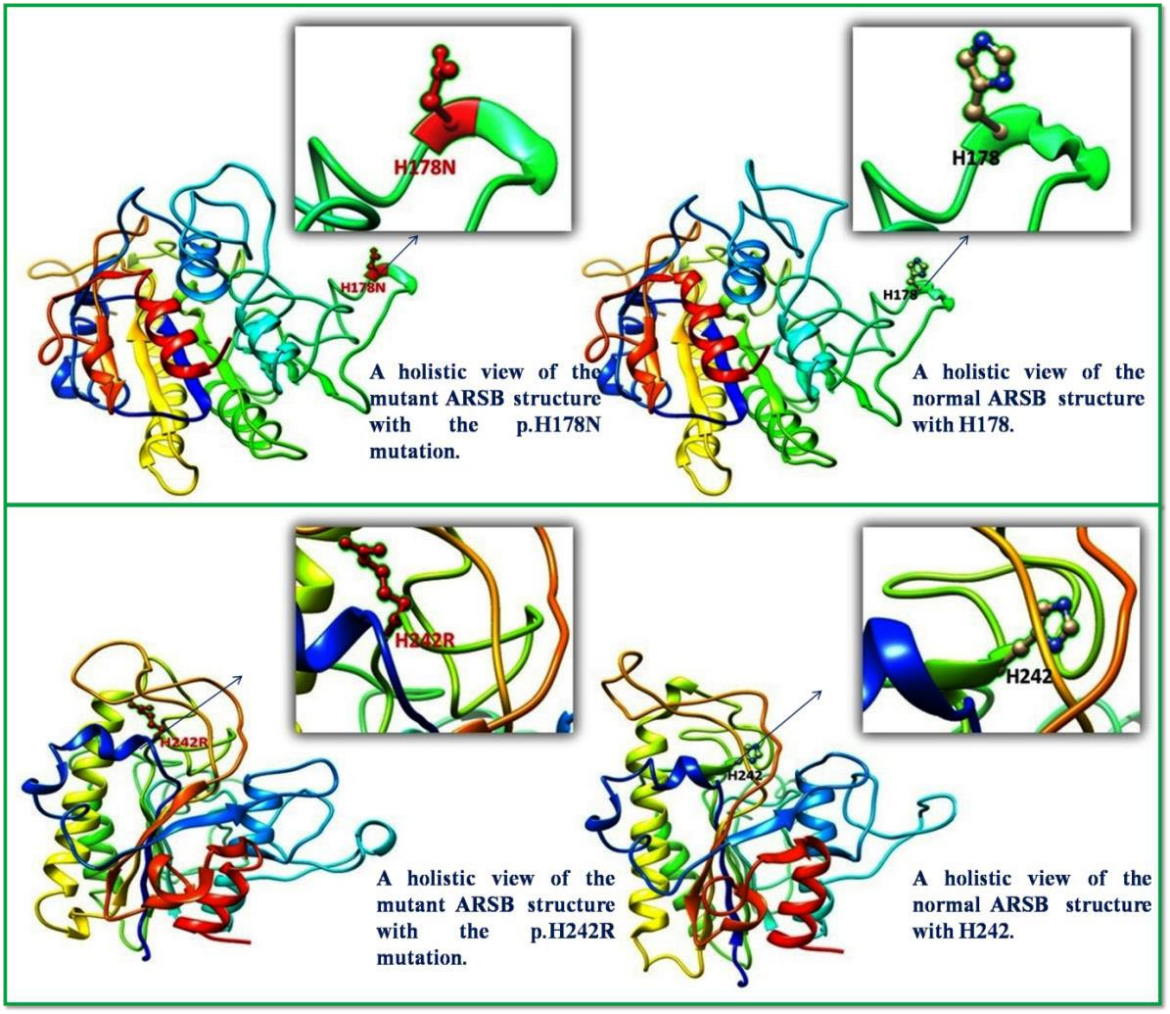
Three-dimensional structure of ARSB; comparison of localization of p.H178N (top) and p.H242R (below) with natural protein. The normal and changed amino acids are represented as a ball-and-stick model

**Table 1:** Sequences of primers used in PCR

Exon	Forward	Reverse	Amplicon (bp)
1	GCAGCCCAGTTCCTCATTCTAT	GCCTGGAAGAGCGAGGTT	554
2	CCTCAAGCCAGTACAGGAATAG	CATTAGAAAGCAGCCCCATTAC	466
3	CCTCGTCACGGGTAATCAAT	ACGCCTAATAAATCACACCCTAG	509
4	CAATGCATTCTGTAGGTTGTCTTG	CTAACCGCTCCAATTTGTCTTC	418
5	TCACACTGGGCCTTACTTATG	ATGGGCTGAGGACAATCTTG	675
6	AACGATGACTGCTTGAAGATG	TGAGATGAGCGGGAGATATG	395
7	AGCTACTGTTCTGCAAGGGTATA	GAGAAATGGCCGTGGGAATC	271
8	CACTAGCTCATGCCCACTAAATG	GACACCTCGGTGTGGTTTAAG	696

**Table 2:** PCR conditions

Exon	Pre-denaturation	Denaturation	Annealing	Extension	Final extension	Cycle
Exon1	95 °C/5 min	95 °C/1 min	63 °C/30 sec	72 °C/1 min	72 °C/5 min	35
Exon2	95 °C/5 min	95 °C/30 sec	63 °C/30 sec	72 °C/1 min	72 °C/5 min	35
Exon3	95 °C/5 min	95 °C/30 sec	63 °C/30 sec	72 °C/30 sec	72 °C/5 min	35
Exon4	95 °C/5 min	95 °C/30 sec	63 °C/30 sec	72 °C/30 sec	72 °C/5 min	35
Exon5	95 °C/5 min	95 °C/30 sec	63 °C/30 sec	72 °C/30 sec	72 °C/5 min	35
Exon6	95 °C/5 min	95 °C/30 sec	63 °C/30 sec	72 °C/30 sec	72 °C/5 min	35
Exon7	95 °C/5 min	95 °C/30 sec	63 °C/30 sec	72 °C/1 min	72 °C/5 min	35
Exon8	95 °C/5 min	95 °C/30 sec	63 °C/30 sec	72 °C/30 sec	72 °C/5 min	35

**Table 3:** Clinical characters and biochemical analysis of MPS VI patients

Family ID	Patient ID	Diagnosis age (years) /gender	${\mathrm{ARSB Activity}}^{a}$	${\mathrm{mg GAGs}/\mathrm{mm Cr2}}^{b}$	${\mathrm{Clinical Features}}^{c}$
F1	P1	3/FM	NI	NI	CC, CCO, CF,CFF, CTS, GR, IH, JS, MC, UH, SK, SCC
F1	P2	2/M	0.47%	NI	CC, CCO, CF, CFF, CTS, GR, IH, JS, MC, UH, SK, SCC
F2	P3	2/M	0.03%	46.3	CC, CCO, CF, CFF, GR, IH, JS, MC, MY, UH, SK, SCC,
F3	P4	3/M	0.02%	40.0	CC, CCO, GR, HM, IH, JS, MC, SK
F4	P5	3/FM	%0.03	NI	CC, CCO, CFF, GR, HM, IH, MC, SK
F5	P6	3 /M	%0.02	59.6	CC, CCO, CFF, GR, HD, IH, MC, UH, SK

a . ARSB activity in the patient's leukocytes (L) or skin fibroblasts (F) refer to the mean value of several controls analyzed in the same experiment.

b . Urine glycosaminoglycans concentration expressed as mg GAG/mmol creatinine. Normal values: 8.01 ± 3.67 (0–1 years); 4.15 ± 2.07 (2–4 years); 3.02 ± 1.6 (4–10 years); 2.76 ± 0.99 (10–15 years); 1.48 ± 0.87 (15–20 years).

c . CC, corneal clouding; CCO, cardiac complications; CF, cardiac failure; CTS, carpal tunnel syndrome; CFF, coarse facial features; GR, growth retardation; H, hepatomegaly; IH, impaired hearing; MC, macrocephaly; JS, joint stiffness; SK, skeletal abnormalities; UH, umbilical hernia; SCC, spinal cord compression; NI, no information.

**Table 4:** Effect of the novel missense substitutions on the function, pathogenicity, and stability of ARSB, as predicted by the use of bioinformatics tools

Effect on the function			Pathogenicity	Stability
Variant	SIFT^𝑎^	POLYPHEN^𝑏^	Mut pred^𝑐^	I −Mut^𝑑^
H242R	Damaging	Probably Damaging	Disease	Decrease
Load Score	0	1	0.935	0.31
H178N	Damaging	Probably Damaging	Disease	Decrease
Load Score	0.02	0.999	0.863	-2.64

Two novel substitutions are likely to be pathogenic, based on predictions by all the tools used.

a . According to SIFT, a mutation is “damaging” if the score is ≤0.05.

b . In PolyPhen-2, the scores for neutral to damaging range from 0 to 1, with 1 being “probably damaging”.

c . In MutPred the score probability ≥0.75 is considered a “very confident hypothesis” for a mutation to be causative of disease.

d . In I-Mut, an amino acid substitution with a score ≤ 0 decreases stability of the protein

***PCR sequencing and mutational analysis of the ARSB gene***

Intronic primers were designed for the *ARSB* gene using Primer3 version 0.4.0 software (Table 1). Genomic DNA was extracted from peripheral blood samples of study participants using the commercial Parstous DNA blood mini kit. For all patients, genomic DNA extracted from whole blood and 100 ng genomic DNA was subjected to PCR for amplification of exons 1–8, including coding regions and the exon/intron boundaries of the *ARSB* gene. PCR conditions were summarized in Table 2. Thereafter, PCR products were subjected to Sanger sequencing by the ABI 3700 capillary sequencer (Macrogen, Korea). Sequencing data were analyzed using the Chromas Lite software and were compared to the genomic reference sequence (NM_000046.4). To predict whether the variations are disease-related, in silico predictions were obtained through the bioinformatics tools SIFT, PolyPhen-2, MutPred, and I-Mutant. Meanwhile, to better understand the structural effects induced by novel missense substitution in the ARSB protein, protein homology modeling was performed using the Chimera software (The Chimera Molecular Graphics System, Version 1.11.2 Schrodinger).

## Results

***Clinical and biochemical findings***

The early onset of disease and both enzyme activity and urinary GAG values associated with clinical presentations indicated a severe form of MPSVI in all 6 patients. Detailed information on clinical protests of patients and enzyme activity are provided in Table 3.

***Analysis of the ARSB gene***

The entire open reading frame of the *ARSB *gene and all exon-intron boundaries were analyzed in six patients by direct sequencing of genomic PCR products. In total, we found four substitutions, three of which were previously unknown: p.H178N, p.H242R, and p.*534W (Figure 1). All patients were born to consanguineous parents and were homozygote for detected variants in the *ARSB* gene (Figures 2 and 3). Also, we found four homozygous polymorphisms; all of them have been previously reported by other studies. The mutations and polymorphisms are represented schematically in Figure 1. 

***Structural effects of the mutations***

Two novel missense substitutions p.H178N and p.H178R are not reported in dbSNPs or the Human Gene Mutation Database (HGMD). Therefore, molecular models of these two novel missense substitutions were compared with the molecular structure of native ARSB (PDB code: 1FSU) using the Chimera software (Figure 4). These mutations affect the three-dimensional structure of the protein and decrease the enzyme’s structural stability, thereby impress the function of the ARSB protein. Specifically, in silico analysis of the enzyme with the p.H178N mutation predicted it to be extremely unstable. Moreover, in silico prediction of the pathogenicity, functional effect, and stability revealed that these variants are presumably disease-causing mutations and cannot be considered polymorphisms (Table 4).

***IVS5+2T>C***

The substitution detected in the two Afghan sibs (P1 and P2), has previously been reported by others (20). This mutation had occurred in the splice donor site at the exon 5–intron 5 junction and altered the 100% conserved GT dinucleotide in the donor splice site of intron 5, which results in skipping of exon 5, protein frameshift, and premature termination.

p.*534W

The novel point mutation p.*534W within translational termination codon, which is found in homozygote form in p4, results in a TGG Tryptophan codon and creates an open reading frame containing 577 amino acid residues, compared with the 533 amino acid residues in natural form. In the p.*534W mutant ARSB polypeptide, the confirmation of the protein is altered by the additional 50 amino acids at the C terminus and seems to encode a catalytically active enzyme. In this patient, the severe deficiency of ARSB enzyme activity is most likely due to an altered three-dimensional structure of the mutant polypeptide, which has an influence on the structure of the protein in solution and a higher susceptibility to proteinases, causing the majority of the mutant precursor to be degraded before reaching the trans-Golgi.

***p.H178N***

Two unrelated individuals (P5 and P6) were found to be homozygous for the same novel p.H178N substitution. Introduction of an Aspargine residue to ARSB structure, may lead to the change in α/β topology of protein and cause misfolding or instability of the enzyme.

***p.V358M***

We found p.V358M polymorphism in P3 to cause a reduction in ARSB activity when accompanied with a disease-causing mutation in the same chromosome (19).

***p.H242R***

The novel substitution p.H242R was found in the homozygous state in P3 associated with causative polymorphism p.V358M. The nonsynonymous substitution p.H242R introduces a positively charged side by Arginine and destabilizes the active site by the elimination of hydrogen bonding between modified His242 and the sulfate ester of the Cys91 (6). In addition, the association of this mutation with p.V358M polymorphism probably increases the severity of the disease.

## Discussion

In homogeneous populations such as Iran’s, which has a high rate of consanguineous marriages, there is an increased rate of autosomal recessive genetic disorders such as MPS VI. MPS VI is a rare progressive lysosomal storage disorder caused by the deficit of the ARSB enzyme. According to international studies, no incidence data for MPS VI in Iran have been published, but information from Iran Healthcare Network indicated that MPS VI is not so frequent in Iran either. In this study, we analyzed four Iranian patients and two patients with Afghan origin and found three novel substitutions, one mutation, and four polymorphisms that were previously characterized.

The IVS5+2T>C substitution is an intronic mutation, which was detected in two patients with Afghan origin. Until now, nearly 10 splice site mutations in the *ARSB* gene have been detected and approximately 50% of these mutations occur in intron 5 (21). c.1142+2T>A mutation, in the donor splice site of intron 5, promotes skipping of exon 5 (19). Homozygosity for c.1142+1G>T in a patient with a very high GAGs resulted in total loss of ARSB protein. Two acceptor splice site mutations c.1143-1G>C and c.1143-8T>G in intron 5 of the *ARSB *gene were identified respectively in 21.9% and 12.5% of mutant alleles from 16 Spanish and Argentinian patients with MPS VI. These mutations cause skipping of exon 6 and premature termination of the protein translation, which result in rapid progressive phenotype with severe manifestations (14, 22).

Among the detected variants, the p.*534W is a nonstop mutation. Most polypeptides resulting from a gene with a nonstop mutation are nonfunctional due to their extreme length and may trigger nonstop mRNA decay (23). A (2011) study of 119 nonstop mutations in 87 different genes, known to cause human inherited disease, identified excess in the range 150–199 nucleotides of downstream in-frame mutated stop codons (24). Researchers reported the p.*534 Q transition in the *ARSB* gene in an MPSVI patient and indicated that the majority of the p.*534Q mutant polypeptides appear to be degraded before reaching the trans-Golgi (23).

The p.H178N substitution was found in two out of our six patients, which connects the α-helix and the following β-strand that are located in the N-terminal domain of the ARSB protein. The vulnerability of the ARSB protein to substitutions in H178L, in the 2007 study by Karageorgos *et al.*, supports the pathogenic nature of p.H178N (14). Also, pathogenicity of 4 missense mutations p.G446R, p.C447S, p.C447F, and p.L472P, which impress connector amino acids between α-helix and β strand can be another reason for pathogenic nature of the substitution found through this study (14, 17).

Among the detected mutations, p.H242R causes a structural change in the active site pocket. The ARSB active site involves at least 10 residues (D53, D54, C91, R95, H147, K145, H242, D300, N301, and K318) (14, 25). Mutation at any of these sites would produce a nonfunctional ARSB protein. The p.N301K mutation, located at the active site, is severely pathogenic with rapidly progressive phenotypes of the homozygotes in which it was first found. p.R95Q is another substitution at the active site, which has been shown to cause a large structural change, mainly in the active site pocket and decrease the activity of the ARSB protein to 0.02% (1, 7). In our study p.H242 in P3, was associated with p.V358M polymorphism. One of the most frequent polymorphisms detected in the MPSVI population is p.V358M; homozygosity for this polymorphism, suggests a co-segregation with disease-causing mutations (26). p.V358M polymorphism is a frequent amino acid substitution among Caucasoids (38% of 220 alleles) and was present in 32% of the 100 alleles of healthy Spanish individuals screened (19). In 2008 Karageorgos *et al.* indicated that p.L72R mutation, together with the p.V358M polymorphism located on the other allele, reduced ARSB activity by up to 16% (26).

## Conclusion

Findings from this study lend support to the hypothesis that missense mutations are the most frequently-reported mutations in MPSVI and are distributed in the whole gene. Also, the rate of parental consanguinity in these six families is 100% (5/5 families). This is surprising, considering that most mutations in the *ARSB *gene are rare and family specific. In this study, two different families had the same substitution p. H178N, the reason for which is not clear. This might be because parents may be distantly related or because certain variants may be more frequent in some communities or geographic locations. In our study, the patients were few and from the same geographic locations. Determining the incidence and prevalence of MPS VI using a cohort study and identification of *ARSB *gene mutations and genotype-phenotype correlations in a larger number of patients would contribute to a better understanding of the MPS VI disorder as a whole.
